# Capture of complete ciliate chromosomes in single sequencing reads reveals widespread chromosome isoforms

**DOI:** 10.1186/s12864-019-6189-9

**Published:** 2019-12-30

**Authors:** Kelsi A. Lindblad, Jananan S. Pathmanathan, Sandrine Moreira, John R. Bracht, Robert P. Sebra, Elizabeth R. Hutton, Laura F. Landweber

**Affiliations:** 1 Departments of Biochemistry & Molecular Biophysics and Biological Sciences, Columbia University, New York, NY 10032 USA; 2 Lewis-Sigler Institute for Integrative Genomics, Princeton University, Princeton, NJ 08544 USA; 3 Department of Biology, American University, 4400 Massachusetts Avenue, NW, Washington, DC 20016 USA; 4 Icahn Institute and Department of Genetics and Genomic Sciences, Icahn School of Medicine at Mount Sinai, New York, NY 10029 USA; 5 Watson School of Biological Sciences, One Bungtown Road, Cold Spring Harbor,, NY 11724 USA

**Keywords:** Ciliate, *Oxytricha*, PacBio, SMRT sequencing, Genome assembly, Alternative fragmentation

## Abstract

**Background:**

Whole-genome shotgun sequencing, which stitches together millions of short sequencing reads into a single genome, ushered in the era of modern genomics and led to a rapid expansion of the number of genome sequences available. Nevertheless, assembly of short reads remains difficult, resulting in fragmented genome sequences. Ultimately, only a sequencing technology capable of capturing complete chromosomes in a single run could resolve all ambiguities. Even “third generation” sequencing technologies produce reads far shorter than most eukaryotic chromosomes. However, the ciliate *Oxytricha trifallax* has a somatic genome with thousands of chromosomes averaging only 3.2 kbp, making it an ideal candidate for exploring the benefits of sequencing whole chromosomes without assembly.

**Results:**

We used single-molecule real-time sequencing to capture thousands of complete chromosomes in single reads and to update the published *Oxytricha trifallax* JRB310 genome assembly. In this version, over 50% of the completed chromosomes with two telomeres derive from single reads. The improved assembly includes over 12,000 new chromosome isoforms, and demonstrates that somatic chromosomes derive from variable rearrangements between somatic segments encoded up to 191,000 base pairs away. However, while long reads reduce the need for assembly, a hybrid approach that supplements long-read sequencing with short reads for error correction produced the most complete and accurate assembly, overall.

**Conclusions:**

This assembly provides the first example of complete eukaryotic chromosomes captured by single sequencing reads and demonstrates that traditional approaches to genome assembly can mask considerable structural variation.

## Background

Whole-genome shotgun sequencing, first pioneered in eukaryotes during the human genome project, has become such common practice that over 38,000 genome assemblies are available from NCBI today [1]. Despite its ubiquity, genome assembly is still a challenge, requiring the computation of overlaps among millions of short reads. In particular, the use of short reads makes it difficult to place repetitive elements, resolve the length of microsatellite repeats, or capture haplotypes over large genomic regions. Traditional whole-genome shotgun sequencing leaves much to be desired for non-model genomes that exhibit either long repeats or high polymorphism rates that fall outside the assumptions of most assembly programs, such as plant genomes that contain high levels of repetitive elements and high ploidy [2, 3], or genomes with large stretches of similarity that result from whole-genome duplications, such as the ciliate *Paramecium* [4]. Despite improvements in assembly algorithms, the best way to completely overcome these issues would ultimately be to use a sequencing method capable of accurately reading the sequence of each chromosome in full. Although current sequencing technologies fall far short of this mark, read lengths have increased substantially. Pacific Biosciences’ single-molecule real-time (SMRT) sequencing platform achieves read lengths as high as 50,000 base pairs [5], while reads over 200,000 base pairs long have been reported from Oxford Nanopore’s MinION [6]. The higher resolution provided by these long reads has made it possible to produce high-quality reference sequences that capture structural variation that short-read sequencing cannot resolve [7, 8] and even automate the completion of microbial genomes [9].

While it is not yet possible to produce reads long enough to capture most eukaryotic chromosomes, *Oxytricha trifallax*’s tiny “nanochromosomes” fall well within the range of recent long-read sequencing technologies and, themselves, offer powerful models for studying eukaryotic chromosome biology [10, 11]. Like all ciliates, *Oxytricha* has two nuclear genomes, a transcriptionally silent germline and a compressed somatic genome used for most of the cell’s transcription. The germline genome has a complex architecture containing > 225,000 short genic sequences (macronuclear destined sequences, MDSs) that assemble during development to form the somatic genome. In addition, approximately 22% of MDSs are present in a permuted order or inverse orientation in the germline, and require descrambling during formation of the somatic chromosomes, together with removal of thousands of noncoding sequences (internally eliminated sequences, IESs) that interrupt MDSs [12]. While the germline genome contains hundreds of long chromosomes, the somatic genome is highly fragmented with ~ 20,000 different chromosomes that average just 3.2 kb in length [13, 14], possess very few well-positioned nucleosomes [10], and derive from a copy of the germline through an elaborate process of RNA-guided genome rearrangement that eliminates 90–95% of the germline sequence, including all IESs, stitches together the remaining germline segments in the correct order [15, 16], and adds telomeres to chromosome ends (reviewed in Yerlici and Landweber [17]).

In addition to small chromosome size, *Oxytricha*’s somatic genome displays several features that complicate traditional genome assembly. Approximately 25% of chromosomes contain one or more internal sites used for telomere addition, which terminates the chromosome. The same proportion of chromosomes use alternative recombination between germline segments. The use of internal telomere addition sites and alternative chromosome fragmentation produces a family of chromosome isoforms that contain only part of another chromosome’s sequence [14]. Furthermore, somatic chromosomes exhibit copy number variation that can range over orders of magnitude, which is well outside the assumptions of most assembly programs and sequencing techniques.

However, while long-read sequencing has the potential to solve many issues associated with the assembly of *Oxytricha*’s macronuclear genome, it also has a major drawback: SMRT sequencing and other long-read technologies produce reads with a much higher error rate than those produced by short-read sequencers. Raw PacBio reads may have up to a 13% error rate, compared to a ~ 0.5% error rate for Illumina [18]. The raw reads therefore require an additional pre-processing “error correction” step prior to assembly. Traditionally, this has been accomplished by aligning short reads to error-containing long reads and using a consensus call method to infer the correct sequence of the long read. The advent of pipelines like PBcR that produce corrected long reads by aligning raw long reads to long reads [5], may eliminate the need for pre-processing correction with short read sequencing but they require much greater PacBio coverage. After self-correction, PacBio reads still exhibit a basal error rate of ~ 2–3% [19], compatible with modern assemblers, but the resulting assembly needs to be further improved with post-assembly correction by short-reads.

In 2013, our lab published a high-quality assembly of *Oxytricha’s* somatic genome using a combination of Sanger, 454 and Illumina data. Here we present an updated version incorporating SMRT sequencing. The improved assembly includes over 13,000 complete chromosomes captured in single reads, entirely without assembly. We find that long reads are ideal for capturing the large number of structural variants in the *Oxytricha* somatic genome and discuss the relative merits of different sequencing strategies for producing the highest-quality assembly for an extensively fragmented genome.

## Results

### Over half the *Oxytricha* somatic genome can be completely sequenced without assembly

**Fig. 1:**
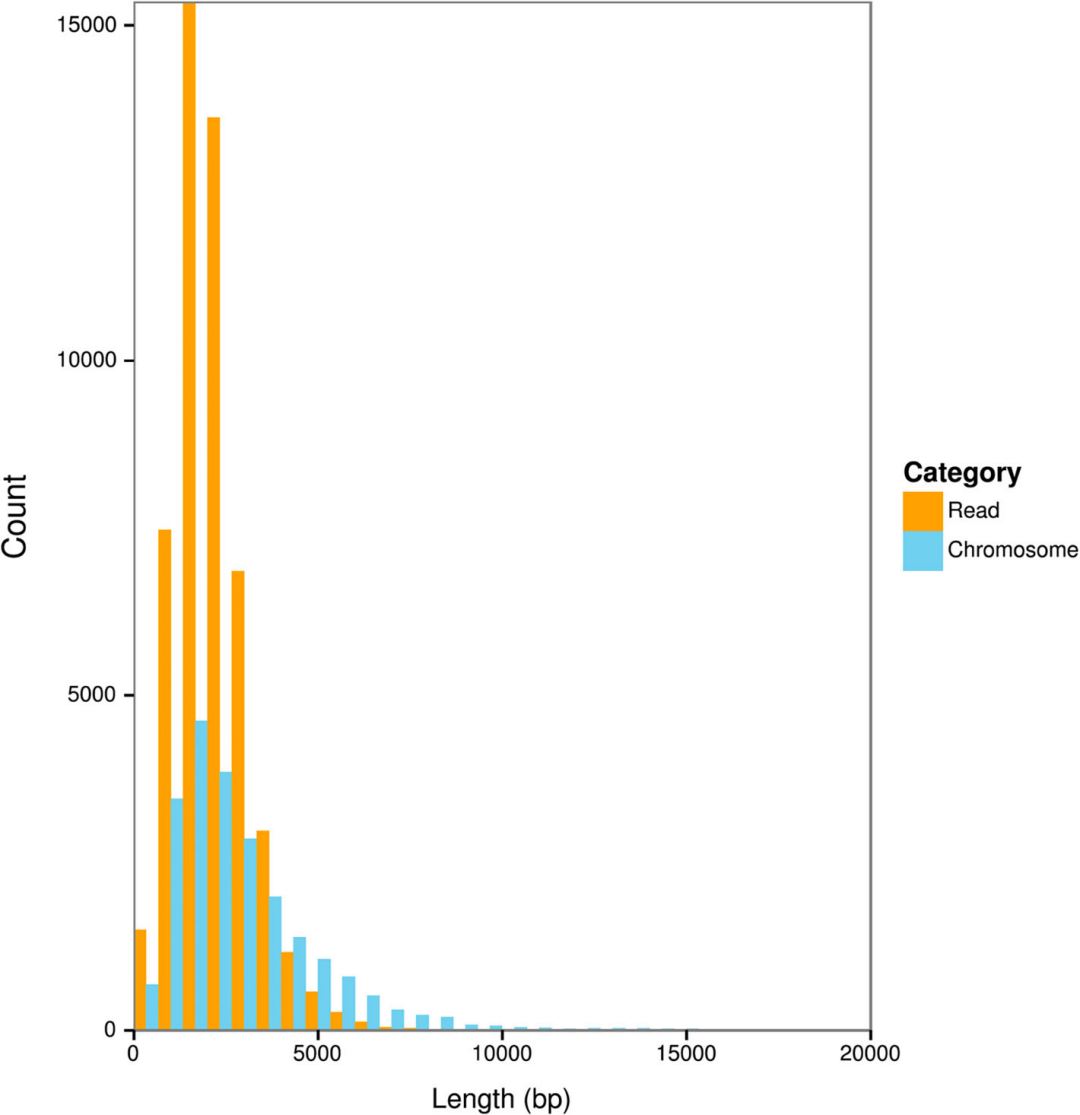
SMRT sequencing reads are long enough to capture complete *Oxytricha* chromosomes. The length distribution of corrected SMRT subreads is similar to the length distribution of *Oxytricha* chromosomes. To improve readability, a random subsample of 50,000 SMRT subreads is shown, and the twelve chromosomes longer than 20,000 bp (from ~ 22,000 bp to ~ 66,000 bp) have been omitted from the plot

**Fig. 2:**
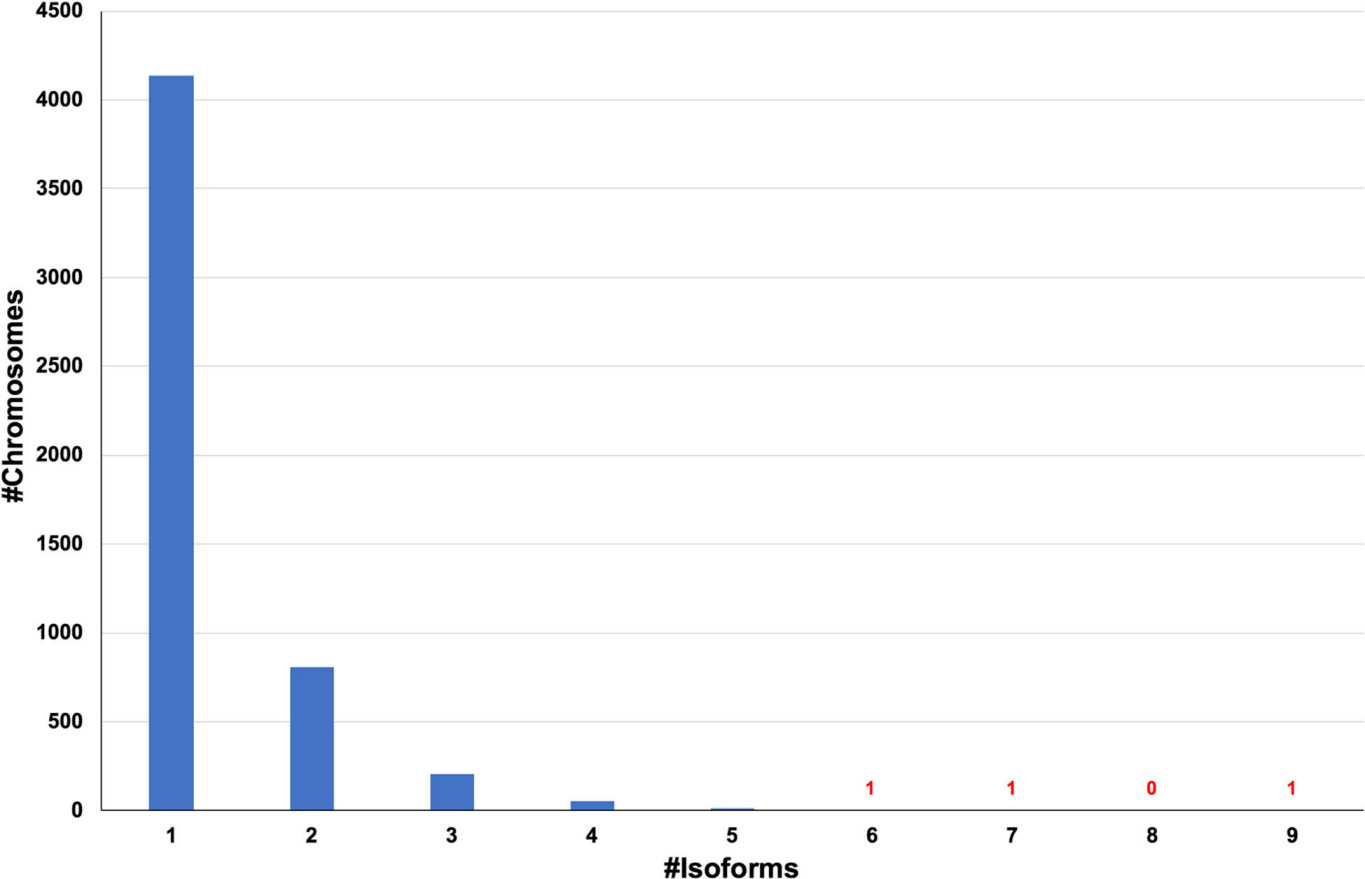
Distribution of the number of isoforms for each chromosome in the hybrid assembly. The distribution of the number of isoforms shows that the majority of the chromosomes have only one isoform. There are few cases with more than one isoform, with a maximum of nine for one chromosome

We isolated *Oxytricha trifallax* strain JRB310 somatic, macronuclear DNA for SMRT sequencing, combining a pilot sequencing run using the P2 chemistry with a second, full run using P3 chemistry, for a total of 10 SMRT cells and 264x genome coverage (Table 1). After filtering and self-correction, we recovered 599,310 reads. As expected, the distribution of sequencing read lengths closely matches the length distribution of *Oxytricha* somatic chromosomes (Fig. 1), and 324,445 corrected subreads contained telomeric sequences on both ends, indicating that they are complete chromosomes. These reads with two telomeres represent 11,378 distinct chromosomes or 51% of the contigs in the published assembly; thus, over half of the genome can be completely sequenced without assembly. We used the Celera Assembler to assemble the corrected reads that lacked telomeric sequences on both ends into contigs and combined these contigs with the single-read chromosomes to produce a long-read-only assembly (Pure PacBio Assembly) (Table 2). Although this assembly contains over 9000 more contigs than the previously published assembly, the majority of the new additions are alternatively fragmented isoforms of previously sequenced chromosomes (Fig. 2 and see “Long-read sequencing discovers novel chromosome isoforms”). While SMRT sequencing provided good coverage of chromosomes around the somatic genome’s mean 3.2kbp length, it was unable to capture most of the shortest chromosomes, largely because short reads (< 300 bp) were filtered out at several points during the data cleaning process. The shortest gene-containing two-telomere chromosome in our assembly was 314 bp, compared to 502 bp in the published assembly. Meanwhile, the longest chromosome captured by a single read was 13,906 bp, which encodes three genes including a Serine/Threonine kinase. Overall, 13% of contigs ≥10,000 bp were present in the long read data, compared to 63% of contigs between 1000 bp and 10,000 bp. This indicates that SMRT sequencing was able to capture long chromosomes in addition to short ones.

**Table 1:** SMRT sequencing of the *Oxytricha* somatic genome

	P2 Chemistry	P3 Chemistry	Combined	Self-corrected
Number of Flow Cells	2	8	10	−/−
Total Subreads	584,388	4,622,662	5,207,050	1,637,578
Total Sequence (GB)	1.37	11.90	13.27	3.5
Mean Read Length (bp)	2350	2575	2545	2152
Max Read Length (bp)	32,258	42,863	42,863	13,629
Genome Coverage^a^	26x	238x	264x	70x

^a^Based on a genome size of 50 MB

**Table 2:** Assembly statistics for long- and short-read genome assemblies

	Published Assembly [14]	Pure PacBio Assembly	Hybrid Assembly
Assembly Size	64 MB	87 MB	108 MB
Total Contigs	22,450	31,664	38,668
Unique Predicted Proteins ^a^	24,963	16,251	27,528
% Identified Busco genes	85%	58%	85%
% Illumina read mapping	96%	85%	95%

^a^ Based on a 90% similarity cut off

To produce a final assembly that combines the strengths of the short read assembly with long read data, we combined our pure long read (PacBio) assembly with high-confidence contigs from the published assembly to create a hybrid assembly containing all high-confidence chromosome isoforms identified by either approach. The hybrid assembly was scrutinized and polished by correcting remaining PacBio sequencing errors with Illumina reads. Of the 38,668 contigs in the final assembly, alternative fragmentation detection revealed 18,617 distinct chromosomes, with 5226 possessing at least one isoform. Note that this number of distinct chromosomes is approximately 2000 more than previously reported in Swart et al. [14]. The previously published genome assembly had been judged largely complete based on its complement of tRNA genes and overlap with the CEG database of core eukaryotic proteins [14]. Here, we measured the completeness of the published and hybrid Assembly using BUSCO [20, 21], both assemblies showed a similar completeness score of 85%. While 100% would be the ideal level of genome completeness expected from BUSCO, this is just one metric for assessing the quality of an assembly. Our lab previously published a study [22] that assembled the somatic genome of six ciliates and assessed completeness using the representation of core eukaryotic genes (CEGs). When we rechecked the completeness of these genomes using BUSCO it produced a range of scores from 70 to 85%. Furthermore, in Chen et al. 2018 [23] the authors used BUSCO to evaluate the completeness of the *Euplotes vannus* genome, as well as the *Oxytricha* and *Tetrahymena* genomes, and observed a similar trend. Moreover, the complement of unique predicted proteins is much higher for the hybrid assembly because we used RNA-seq data from vegetatively-growing, starved, and encysted *Oxytricha* cells for gene prediction with Augustus. The domain analysis of these proteins shows that the hybrid assembly contain only 94 more protein domains that were not identified in the previous assembly. This suggests that, rather than having missed large numbers of functional proteins in the previous assembly, the larger proteome size in the hybrid assembly is mostly accounted for by the presence of variants of existing proteins. Also, while approximately 13,500 new chromosome variants were identified in the long read data, only two entirely new, incomplete chromosomes were discovered. This suggests that the hybrid assembly is virtually complete.

### Long-read sequencing discovers novel chromosome isoforms

**Fig. 3:**
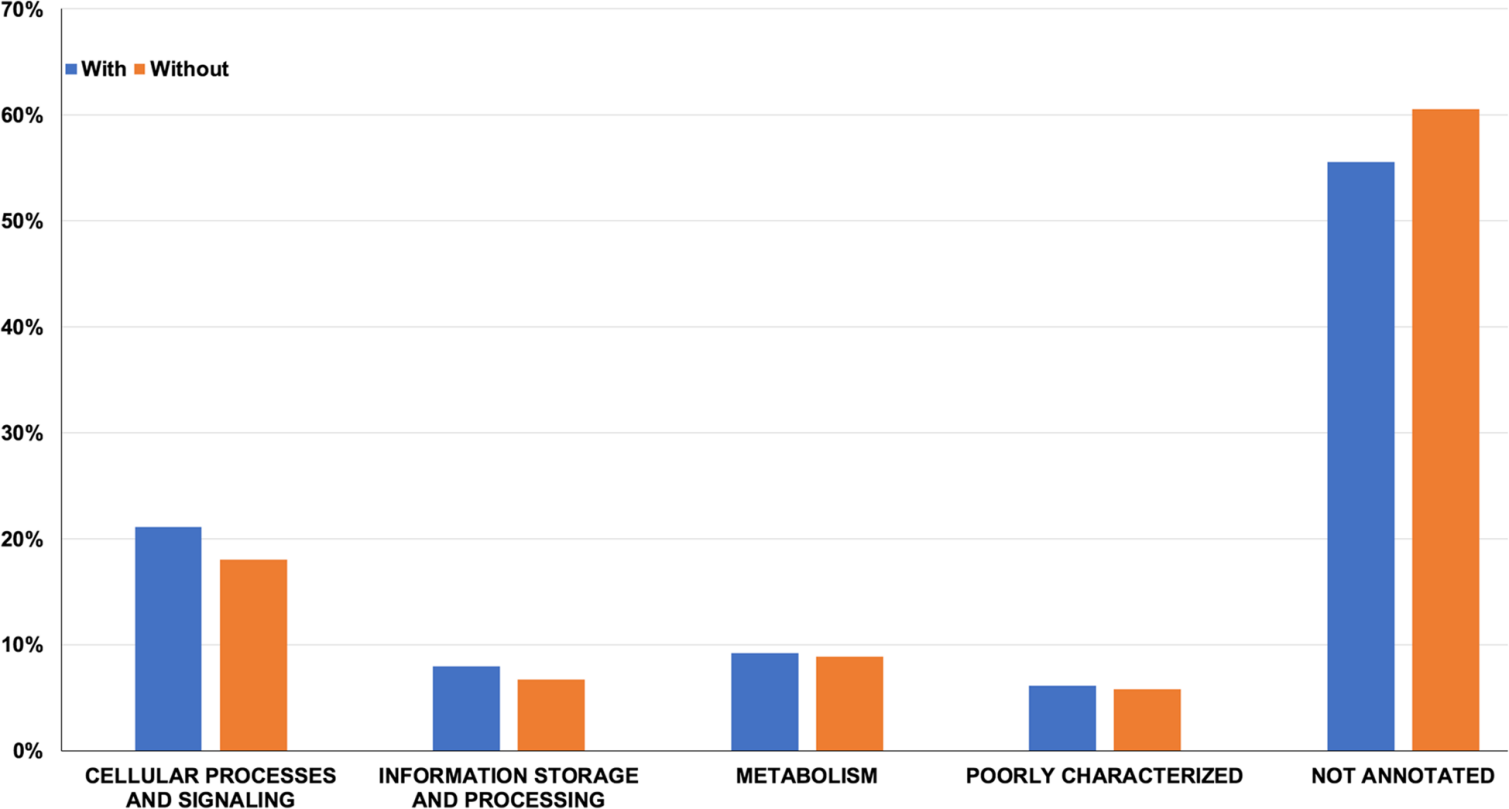
Chromosomes with alternative fragmentation encode for proteins involved in cellular processes and signaling functions. Functional analysis using the KOG database revealed that the encoded proteins on chromosomes with isoforms (blue) are enriched in functions belonging to the cellular processes and signaling category (Fisher test, *p*-value < 0.05). Curiously, the proteins encoded on chromosomes without isoforms are mostly unannotated

*Oxytricha*’s somatic chromosome isoforms are often masked by genome assembly pipelines that merge short chromosomes into larger ones with the same sequence. Previous estimates of the level of alternative fragmentation in *Oxytricha* were based either on PCR examination of individual loci [24] or on the inference of telomere addition sites by identifying pileups of telomere-containing reads [14]. SMRT sequencing captures these variants in their entirety. Our genome-wide analysis of alternative fragmentation sites identified 25,312 distinct chromosome variants, with 5226 of the 18,617 (28%) detected chromosomes demonstrating at least one alternative fragmentation site (Fig. 2). The functional analysis of the proteins encoded by these chromosomes with isoforms suggests that they are mostly involved in cellular processes and signaling functions (Fig. 3). They are enriched in three KOG functional categories: “T” (Signal transduction mechanisms), “O” (Posttranslational modification, protein turnover, chaperones) and “U” (Intracellular trafficking, secretion, and vesicular transport). Curiously, chromosomes that lack isoforms in our study display an excess of predicted proteins with unknown functions.

Mapping telomere-containing Sanger sequencing reads predicted 6695 isoforms [14], 76% of which are also found among the isoforms in the long read assembly. This indicates that SMRT sequencing captures the same kind of isoforms, but notably it finds more of them.

**Fig. 4:**
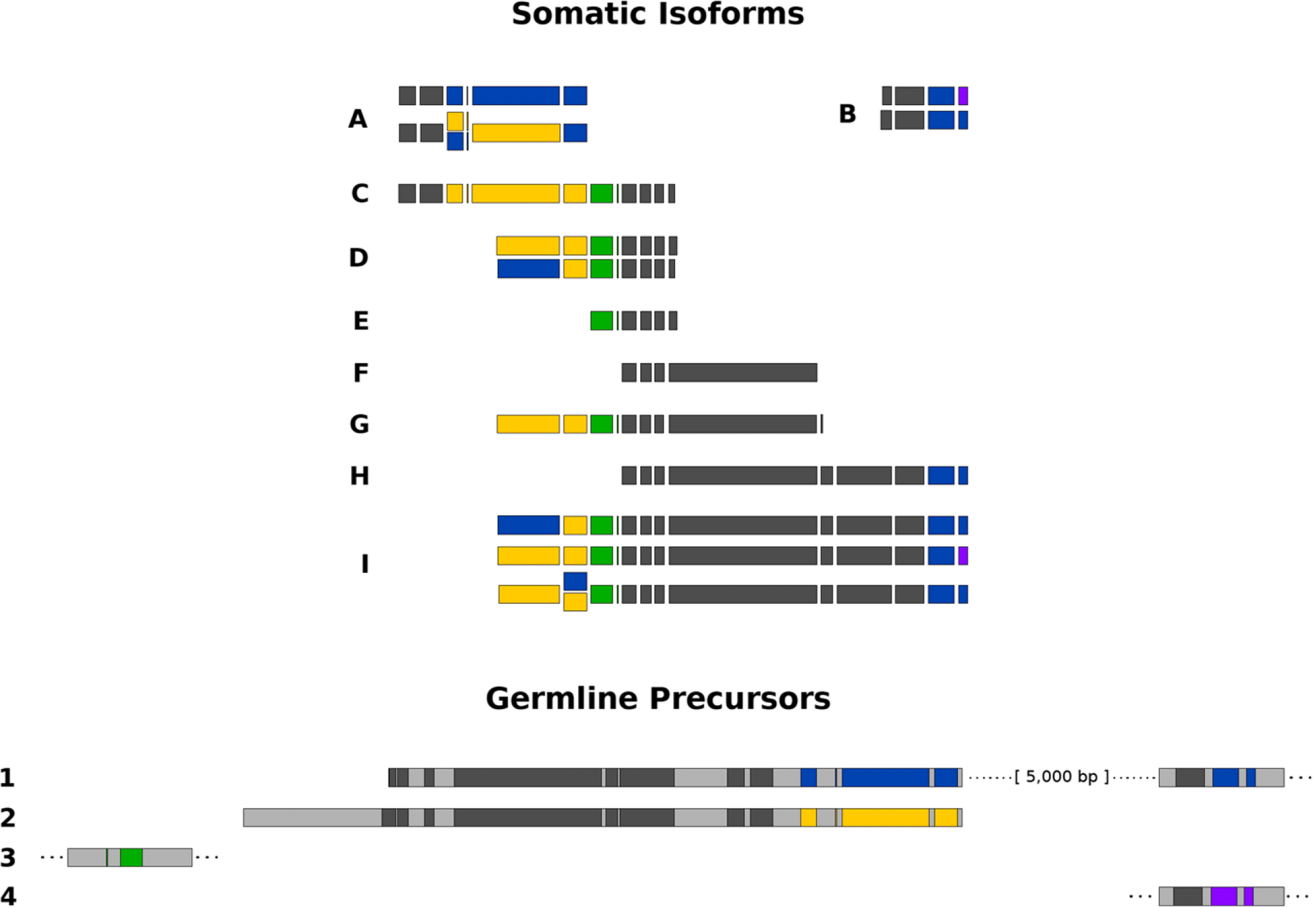
Long-read sequencing reveals underlying structural variation among chromosomes. Segments of four germline contigs (1–4) rearrange to produce nine high-confidence isoforms (A-I) of one somatic chromosome, Contig14329.0. The four germline contigs most likely represent two paralogous loci; contig 2 terminates in repetitive sequences at both ends. These match repetitive sequence at the 3′ end of contig 3 and the 5′ end of contig 4. Dotted lines indicate that a contig extends beyond the region shown. Dark gray blocks on the germline contigs represent somatic sequence that is 100% identical between the two paralogs, while colored sequence represents regions that differ between the two germline loci. Colored segments in the somatic isoforms indicate the corresponding germline segments of origin; two boxes stacked vertically indicate ambiguity when the germline paralogs are identical. While most isoforms contain only sequence from one locus or the other, isoforms “A,” “B,” “D,” and “I” have variants that incorporate sequence from both loci, suggesting that alternative fragmentation and assembly can recombine segments from multiple loci, in addition to variable retention of segments within a single locus

Furthermore, the ability of long read sequencing to retrieve complete sequences of the isoforms, not just their lengths, allows us to examine alternative fragmentation on a genome-wide scale. Previous studies have inferred that multiple germline loci may contribute to families of alternative fragmentation isoforms [24, 25]. With the current data provided by long-read sequencing, we find that some isoforms may derive from mixing and matching between these different loci, rather than from processing each locus separately. Figure 4 shows one of the chromosomes with the most fragmentation isoforms in our dataset, Contig14329.0, that has nine isoforms. Of these, four incorporate sequence from two separate germline loci, suggesting that alternative fragmentation and assembly can recombine segments from multiple loci, which would require inter-chromosomal recombination. The other five isoforms include segments from only one locus or the other. To produce the full complement of isoforms for this chromosome the cell must therefore undertake variable processing within a single locus, as well as combine sequences from multiple loci. Several of the alternatively fragmented isoforms also contain segments from just one locus or the other, suggesting that an unknown mechanism might regulate which isoforms a locus produces. We find that variable processing is widespread, with 2522 out of 5226 (48%) alternatively fragmented chromosomes deriving from two or more paralogous germline loci. Moreover, it will be illuminating to mine the data for evidence of interallelic rearrangements in *Oxytricha’s* somatic genome. However, we found that the current data and methods were insufficient to phase each chromosome from the hybrid assembly to produce a high quality haploid version of the genome assembly.

### Hybrid error correction produces the most complete somatic genome assembly

**Fig. 5:**
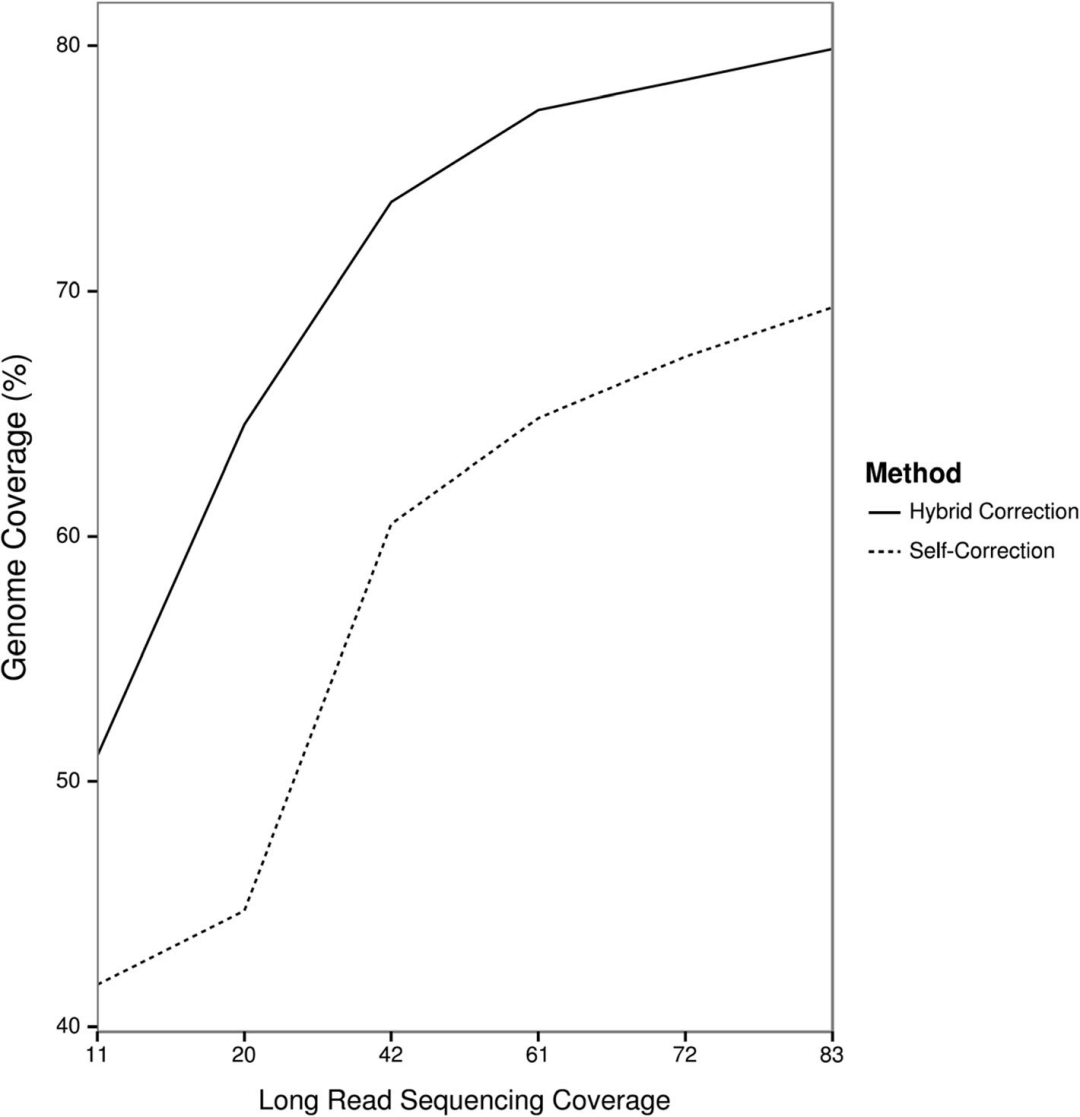
Hybrid error correction outperforms long read self-correction. With 50x coverage of short-read data, hybrid error correction produces a more complete assembly than self-correction, even at twice the minimum recommended long-read coverage

To determine whether pure long-read sequencing produces an assembly of similar quality to a hybrid strategy that uses short reads to correct PacBio reads, we subsampled our long read data and assessed the completeness of assemblies produced using the two correction methods. Overall, hybrid error correction outperforms long-read-only error correction at all sequencing depths (Fig. 5), and while the number of contigs recovered by hybrid error correction begins to saturate with eight flow cells’ worth of data, the steep slope of the long-read-only curve suggests that considerably more sequencing depth would be necessary to correct all chromosomes using only long reads.

The heterogeneous copy number of chromosomes in *Oxytricha*’s somatic genome may be the root cause for the inadequacy of long-read error correction. While the average somatic chromosome copy number is approximately 2000n [13], some chromosomes can be amplified to over 200,000 copies. This reduces the effective coverage for low-copy number chromosomes, as more abundant chromosomes absorb a disproportionate amount of sequencing depth. Both the hybrid-corrected and self-corrected genome assemblies were biased towards chromosomes with significantly greater copy number than average read coverage, even when all flow cells were incorporated (Welch’s one-sided *t*-test, *t* = 4.1652, *p* = 1.559e-05 for hybrid correction, *t* = 4.7637, p = 1.559e-05 for self-correction). However, hybrid error correction resulted in a steeper decline in mean chromosome copy number across the genome as sequencing depth increased, compared to self-correction (79 fewer for the hybrid error correction, 13 fewer for self correction). This indicates that the hybrid error correction incorporated more low-abundance chromosomes as the amount of long read data increased, relative to the self-correction method. While we recovered 135x coverage of corrected sequence from strictly long reads, this derived from only hundreds of thousands of PacBio reads, each an individually sampled molecule, compared with tens of millions of short-read Illumina sequences. The increased depth that can be achieved with short reads is thus more important to the completeness of the final genome assembly than the increased resolution provided by long-read sequencing.

## Discussion

As long-read sequencing technology improves, it may eventually be possible to sequence complete chromosomes of most organisms in a single contiguous read. For now, *Oxytricha*’s highly fragmented genome provides the first opportunity for genome sequencing without assembly. This approach permitted the discovery of structural chromosome isoforms that were masked by traditional assembly pipelines. Long-read sequencing dramatically increased the number of alternative isoforms that we could identify. Furthermore, we conclude that many of these isoforms may derive from recombination among multiple germline loci, in addition to variable processing within a locus. Where allelic variation is present, this implies that genome rearrangement may occur between—as well as within—germline chromosomes. The observation that some isoforms derive exclusively from one locus or the other also raises the question of what regulates this selection and what determines the range of isoforms produced. The finding that piRNAs can strongly influence chromosome fragmentation patterns and lead to alternatively processed chromosomes [26] suggests that the piRNA pathway is involved [27, 28] in this process.

The long read sequencing in this study permitted a higher quality examination of closely-related chromosome isoforms than the previously published assembly. However, for de novo genome assembly, the variation in chromosome copy number in *Oxytricha* makes the approach less economical than short-read sequencing. Alternatively, for species that possess highly fragmented genomes and gene-sized chromosomes with variable chromosome copy number, the challenge of genome assembly is similar to transcriptome assembly. As such, the PacBio isoform sequencing (Iso-Seq) pipeline for transcriptome assembly could also be modified for genome sequencing, just as it is capable of capturing complete transcripts without assembly and identifying novel genes and isoforms produced via alternative splicing [29–32]. Overall, we recommend that future studies perform an initial assembly based on short read data and use long reads to investigate structural variants, the area where we reaped the most benefit for this genome.

## Conclusions

The combination of high coverage long and short reads permits the most complete assembly of a ciliate genome, together with the discovery of novel structural variants. The improved *Oxytricha trifallax* macronuclear genome assembly presented here will allow further investigation of chromosome rearrangements in this species and lineage.

## Methods

### Cell growth and culture

Cell growth, harvest, and nuclei isolation of *Oxytricha trifallax* strain JRB310 were carried out as described in [12], with the exception that the pellet was collected after the initial centrifugation step rather than from the 10% gradient fraction to isolate macronuclei rather than micronuclei.

### Library preparation and sequencing

Library preparation and sequencing were per the manufacturer’s instructions for P5-C3 and P6-C4 sequencing enzyme and chemistry, as previously described [12]. Aliquots of 5 μg of extracted high-quality genomic DNA were enriched for MAC DNA and verified using Qubit analysis. DNA was quantified and diluted to 150 μL in Qiagen elution buffer (33 μg/μL). The sample was pipetted into the top chamber of a Covaris G-tube spin column, gently sheared 60 s, 4500 rpm in an Eppendorf 5424 bench top centrifuge, followed by 0.45X AMPure XP purification. ~ 1.2 μg of this sample was used in library preparation exactly as described in [12].

After library preparation, samples were validated as ~ 5 kb via an Agilent DNA 12000 gel chip. Blue Pippin 0.75% agarose cassettes (Sage Science) were used to prepare a MAC-enriched library (5000 bp – 50,000 bp). In 2014 we sequenced two SMRT Cells as a proof of concept. For these initial SMRT Cells the polymerase-template complex was bound to the P5 enzyme using a ratio of 10:1 polymerase to SMRTbell at 0.5 nM, 4 h, 30 °C, then incubated at 4 °C prior to magbead loading and sequencing with the C3 chemistry. In 2015 we sequenced 8 additional SMRT Cells to have enough material for long read self-correction. For these additional SMRT Cells the complex was bound to the P6 enzyme and sequenced using the C4 chemistry. The magnetic bead-loading step was conducted at 4 °C for 60 min. The magbead-loaded, polymerase-bound SMRTbell libraries were placed onto the RSII machine at a sequencing concentration of 100 to 110 pM and sequenced across two SMRT Cells using P5-C3 and 8 additional SMRT Cells using P6-C4 chemistry.

### Genome assembly

We used Pacific Bioscience’s SMRT Pipe 2.3.0 [33] to quality trim and to filter raw SMRT sequencing reads, using default parameters but enabling the artifact filter (parameter value − 1000) in order to remove chimeric reads. Reads that passed the filter were self-corrected using PBcR (default parameters) [5].

Error correction deleted the telomeres from most reads, so we gathered all raw reads that had at least one telomere, based on matching to the regular expression [TG]*TTTTGGGGTTTT, [TG]*GGGGTTTTGGGG, [AC]*AAAACCCCAAAA, or [AC]*CCCCAAAACCCC with an edit distance of two. The first and last 1000 bp of these reads were corrected using ECTools (default parameters) [34, 35] and a 50x coverage subset of Illumina reads from the previously published *Oxytricha* somatic assembly [14]. Chromosome ends corrected in this manner were aligned to the PBcR-corrected read and the missing bases filled in from the ECTools corrected read.

Some corrected reads were chimeras of multiple chromosomes, characterized by embedded telomeric sequences, or sequencing artifacts composed almost exclusively of homopolymer runs. As a result, we filtered out all corrected reads containing a homopolymer run of > 10 bp or a non-terminal telomeric sequence (matching the regular expression [AC]*(CCAAAACCCCAAAA) or (GGTTTTGGGGTTTT)[TG] with an edit distance of one or [AC]*CCCAAAACCCCGGGGTTTTGGG[TG*] or [TG]*GGGTTTTGGGGCCCCAAAACCC[AC*] with an edit distance of three).

After filtering, all reads with telomeric sequences on both ends were considered complete chromosomes and retained, while reads with one or fewer telomeres were assembled using Celera Assembler 8.3rc [5]. We combined the assembled contigs with the two-telomere reads and clustered the resulting sequences at a 90% identity threshold using VSEARCH [36] and took the centroid contig for each of the resulting clusters to produce a final set of unique chromosomes.

We removed duplicated sequences with BBTools dedupe.sh script [37]. We polished our assembly by recursively applying Pilon [38], an error correction tool that uses Illumina reads to correct PacBio sequencing errors.

We determined alternative fragmentation isoforms by extracting all two-telomere single reads and contigs from our data and masking the telomeres according to the procedure described in [14]. We then used BWA MEM [39] to map the masked reads against the subset of unique chromosomes in our assembly. We grouped all reads with both start and end positions within 50 bp of one another into distinct isoforms and clustered all reads assigned to each isoform at a 97% similarity threshold. We added the consensus sequence of each cluster comprising at least two contigs to the assembly.

To finalize the assembly, we added contigs that were captured in the published *Oxytricha* assembly but not in our long-read assembly. These included two-telomere contigs shorter than 600 bp long and contigs either without an analog in the long-read data, or where the longest isoform in the long-read assembly was at least 75 bp shorter than the version in the published assembly. In cases where the published contig was longer and the long-read version had both telomeres, we considered the long-read form an alternative fragmentation isoform and retained it in addition to adding the longer published contig. If the long-read form had fewer than two telomeres, it was discarded instead. Finally, we removed contigs where at least 50% of the contig sequence was covered by a known germline repetitive element or satellite repeat. We also removed as likely contaminants any contigs without any telomeres and which were less than 20% covered in the germline genome.

### Analysis of alternative chromosome fragmentation

To compare the alternative fragmentation isoforms found by SMRT sequencing with those predicted by older sequencing technologies, we masked all two-telomere corrected reads as described above and mapped them against the published somatic genome assembly [14]. We then grouped reads into distinct isoforms as described above, choosing only the longest hit for each read. In addition, because a size selection step was used in the Sanger sequencing that produced the original predicted isoforms, we filtered the resulting isoforms to include only those less than 6000 bp long. To determine whether an isoform found by one method was also discovered by the other, we used BEDTools 2.25.0 intersect [40] with the options -F 90 -f 90 to count only isoforms that were at least 90% covered in both assemblies.

To analyze how somatic isoforms relate to their germline loci, we selected all isoforms supported by at least two corrected reads and aligned them to the germline genome [12] with Megablast [41]. Isoforms containing sequence from more than one paralogous locus were identified by choosing the best hit for each germline sequence comprising the isoform, then filtering for isoforms containing segments from two or more different germline loci.

### RNA-Seq

We prepared RNA-seq libraries from vegetatively-growing, starved, and encysted *Oxytricha* cells. The vegetative culture was grown according to the same procedure used for collecting MAC DNA. Cells for starved and cyst libraries were placed in a clean dish and incubated at 4 °C and room temperature, respectively, for 5 days. RNA for the starved and vegetative samples was extracted using TRIzol® Reagent (Life Technologies™). RNA for the encysted sample was extracted using 0.25 mm silica carbide beads in the UltraClean Microbial RNA Isolation Kit (MO Bio). Three replicates of vegetative cell RNA, three replicates of encysted cell RNA, and one replicate of 4 °C-starved RNA were prepared with the Epicentre Stranded kit, along with a no-RNA input control. cDNA samples were amplified in 12 PCR cycles. Library preparation and sequencing was performed by the Lewis-Sigler Institute for Integrative Genomics Sequencing Core Facility using the Illumina Truseq Library Prep Kit.

### Gene prediction

We used a gene prediction model trained on *Oxytricha* data and presented in [14] in conjunction with AUGUSTUS 3.3.1 [42] to predict genes for all three assemblies. We used the RNA-seq data collected from vegetatively-growing, starved, and encysted cells; previously-published RNA-seq collected from cells undergoing conjugation and genome rearrangement collected from vegetatively-growing, starved, and encysted cells; (at 0, 10, 20, 40, and 60 h after cells were mixed to initiate mating); and transcription start site data [10] to provide hints to the gene prediction software. We mapped reads to the genomes using HISAT2 v2.0.5 [43], then generated hints files according to the instructions on the AUGUSTUS web site [44] . We ran AUGUSTUS with the options --UTR = on and --alternatives-from- evidence = true. We annotated the proteins using PANNZER2 [45] using default parameters and predicted protein domains using Interproscan 5 RC5 [46] using default parameters.

### Subsampling analysis

We took random subsets of one, two, four, six, seven, and all eight of the flow cells from the 2015 sequencing run and used them to complete de novo *Oxytricha* assemblies. The reads were first filtered using the same methodology used for the primary assembly, then error corrected using either the PBcR pipeline or ECTools. For the one- and two-flow cell subsets corrected by PBcR, we used the recommended high-sensitivity parameter settings intended for low coverage assemblies (QV = 52 asmOvlErrorRate = 0.1 asmUtgErrorRate = 0.06 asmCgwErrorRate = 0.1 asmCnsErrorRate = 0.1 asmOBT = 1 asmObtErrorRate = 0.08 asmObtErrorLimit = 4.5 utgGraphErrorRate = 0.05 utgMergeErrorRate = 0.05). Otherwise, all settings used were the default. After error correction, reads were assembled using Celera assembler. To assess genome completeness, we mapped corrected reads and assembled contigs against the previously published *Oxytricha* assembly and counted the number of contigs at least 80% covered by either a single read or a single contig from the de novo assembly.

### Statistical analysis

We carried out all statistical analyses in the R programming environment [47] and used the ggplot2 package [48] to generate figures.

## Data Availability

All sequencing data used in this supporting the conclusions of this article are available to the public. DNA sequencing reads used for error correction can be accessed from the Short Read Archive (http://www.ncbi.nlm.nih.gov/sra) under accession no. SRX190400. The complete hybrid PacBio assembly has been deposited at DDBJ/ENA/GenBankunder the accession AMCR00000000. The version described in this paperis version AMCR02000000 (under BioProject PRJNA74629), also available at http://knot.math.usf.edu/data/external/dataMAC310/pacbio_mac_final.fa (this assembly replaces the previous short read-based assembly). The pure PacBio assembly is available at http://knot.math.usf.edu/data/external/dataMAC310/pacbio_pure_final.fa. The raw PacBio sequencing reads (SRX2335607 and SRX2335608) and vegetative (SRX2354037), starved (SRX2354036), and encysted-cell (SRX2354038) RNA-seq reads used for gene prediction are available at NCBI under BioProject PRJNA352762.

## Abbreviations

BUSCO: Benchmarking Universal Single-Copy Orthologs
CEG: Core Eukaryotic Genes
SMRT: Single-Molecule Real-Time
